# Industry-Sponsored Research Payments to Gastroenterologists and Hepatologists in the United States Between 2014 and 2021

**DOI:** 10.7759/cureus.48449

**Published:** 2023-11-07

**Authors:** Anju Murayama

**Affiliations:** 1 School of Medicine, Tohoku University, Sendai, JPN; 2 Department of Population Health Science and Policy, Icahn School of Medicine at Mount Sinai, New York City, USA

**Keywords:** clinical research education, financial conflicts of interest, conflicts of interest, united states of america, united states, sunshine act, physician payment sunshine act, open payments database, industry payment, research payment

## Abstract

Aim

This cross-sectional study aimed to examine the scale and trends of industry-sponsored research payments to gastroenterologists and hepatologists in the United States from 2014 to 2021.

Methods

Using the Open Payments Database and the National Plan and Provider Enumeration System (NPPES), the study analyzed both grant and research payments made to individual gastroenterologists and associated payments made for research where gastroenterologists and hepatologists served as principal investigators.

Results

After adjusting for inflation, the study found that a total of $1.5 billion was allocated to gastroenterologists by 284 companies during this period. Only 15.9% of the 20,986 gastroenterologists received at least one research payment, with associated research payments accounting for 97.6% of all payments. The study also revealed a significant increase in the number of gastroenterologists receiving associated research payments and a more than twofold increase in payments for registered clinical trials from 2014 to 2021.

Conclusion

The healthcare industry allocated large amounts of research funding to gastroenterologists and hepatologists. The study underscores the critical role of industry-sponsored clinical trials in advancing gastroenterological research and treatments.

## Introduction

Over the past two decades, there has been a significant increase in research investment and breakthrough treatments in the field of gastroenterology. This progress has been facilitated by both public sector funding and collaborations with the healthcare industry, which have become increasingly vital for advancing gastroenterological treatments [1,2].

However, a study conducted by Ying et al. revealed that only 6% of all payments made to gastroenterologists, amounting to $27.5 million, were allocated for research and grants in the United States between 2014 and 2020 [3]. This figure may underestimate the true extent of research payments provided to gastroenterologists and hepatologists in the healthcare industry. Previous studies have indicated that many healthcare companies channel their research payments to physicians through teaching hospitals and research institutions [4-13]. For example, the healthcare industry spent over $799.9 million on pulmonologists for research purposes in the United States [4]. Another research showed that the healthcare industry spent more than $1.4 billion on endocrinologists for research purposes in the United States between 2014 and 2022 [11]. Furthermore, given that industry-sponsored clinical trials conducted by physicians are crucial for introducing new treatments to the market, it is important to evaluate the whole magnitude of industry-sponsored research payments to gastroenterologists in the United States. This article was previously posted to the Authorea preprint server on June 20, 2023 [14].

## Materials and methods

Study design and data collection

This cross-sectional study examined the scale and trends of industry-sponsored research payments to all gastroenterologists and hepatologists in the United States using the Open Payments Database from 2014 to 2021. To improve transparency in financial relationships between the healthcare industry and healthcare professionals in the United States, the Physician Payments Sunshine Act was enacted as a part of the Affordable Care Act in 2010. Following the act, the Centers for Medicare and Medicaid Services mandates all pharmaceutical and medical device companies to report their financial transfers exceeding $100 in aggregate annual amounts or $10 per payment to physicians, nurse practitioners, and teaching hospitals from pharmaceutical and medical device companies since 2013. The reported payment data have been published on a federal online database, namely the Open Payments Database, since August 2013 [4,10,15,16]. 

In this study, all physicians whose primary specialty is classified as either gastroenterology, hepatology, transplant hepatology, pediatric gastroenterology, or pediatric transplant hepatology were extracted from the National Plan and Provider Enumeration System (NPPES) database, as previously noted [4,5,15]. Then, all research payments and general payments for grant purposes to the eligible gastroenterologists and hepatologists were collected from the Open Payments Database from January 2014 to December 2022. As payment data in 2013 were a partial disclosure of payments between August and December 2013 and contained many errors, as previously reported [4,17], this study did not collect the payments to gastroenterologists and hepatologists in 2013. This research analyzed both grant and research payments directly made to individual gastroenterologists and research payments made to gastroenterologists serving as principal investigators via teaching hospitals and other research organizations (hereafter associated research payments) [4-6,10]. 

Data analyses

This study conducted descriptive analyses on the extracted payment, including the average, standard deviation (SD), median, and interquartile range (IQR). Per-physician payments were calculated among physicians who received payments, as the majority of gastroenterologists did not receive research payments from the healthcare industry. The payments were analyzed by payment year and content of payments. For associated research payments, per-physician payments were calculated as total payments divided by the number of principal investigators in a research payment, and total research payments were calculated by the number of gastroenterologists times the payment per principal investigator because some research payments included principal investigators with other specialties. 

Furthermore, the study assessed yearly trends in the number of gastroenterologists receiving payments and per-physician payments using population-averaged generalized estimating equations (GEE) at the individual physician level, as noted previously [4,5,18-21]. The annual number of physicians receiving payments and per-physician payments were examined using log-linked GEE with a Poisson distribution and negative binomial GEE, respectively. Inflation in US dollars was adjusted to 2021-dollar values using a Consumer Price Index inflation calculator of the U.S. Bureau of Labor Statistics [16,22,23].

Ethical clearance

Institutional board review and approval were not required for this study, as the study was designed as a non-human subject study of publicly available data.

## Results

After adjusting for inflation, a total of $1,527,127,494 was allocated to gastroenterologists as research payments by 284 companies between 2014 and 2021. Among the 20,986 gastroenterologists included in the study, 3,338 (15.9%) received at least one grant, direct research, or associated research payment during the eight-year period. Associated research payments accounted for 97.6% of all research payments, totaling $1,491.1 million (Table 1). There were large differences between the median and average per-physician payment amounts. The median per-physician combined payment over the eight years was $85,080 (interquartile range: $19,899-$382,206), while the average per-physician payment was $540,848 (standard deviation: $2,309,907) for associated research payments.

**Table 1:** Industry-sponsored grants, direct research, and associated research payments to gastroenterologists between 2014 and 2021 Legend: A) Inflation in US dollars was adjusted to its 2021 value using the Consumer Price Index Inflation Calculator of the U.S. Bureau of Labor Statistics. B) Per-physician payments were calculated among physicians receiving payments. *p<0.001. 95% CI: 95% confidence interval, IQR: interquartile range, SD: standard deviation

Variables	Payment year								Overall	Relative average annual percent change (95% CI), %
	2014	2015	2016	2017	2018	2019	2020	2021		
Direct research payments and grant payments										
Total payment amounts, $^a^	3,320,621	9,396,794	3,195,554	3,488,001	3,284,644	5,185,346	3,766,893	4,372,003	36,009,857 (2.4)	–
Number of physicians with payments, n (%)	256 (1.2)	465 (2.2)	326 (1.6)	327 (1.6)	311 (1.5)	463 (2.2)	336 (1.6)	336 (1.6)	1577 (7.5)	1.0 (-0.7 – 2.8)
Payments per physician, $^a,b^										
Median (IQR)	1,672 (458–8,092)	4,107 (1,143–17,149)	2,404 (866–6,774)	3,211 (375–8,291)	1,799 (707–8,768)	1,788 (612–8,409)	2,849 (814–11,574)	4,011 (1,142–12,842)	4,486 (1,080–21,405)	-3.7 (-10.3 – 3.3)
Average (SD)	12,971 (36,768)	20,208 (109,817)	9,802 (24,611)	10,667 (24,695)	10,562 (22,884)	11,199 (34,772)	11,211 (20,428)	13,012 (29,631)	22,834 (83,286)	
Associated research payments										
Total payment amounts, $^a^	185,784,945	177,006,324	211,589,680	205,089,992	164,522,806	208,984,392	186,044,913	152,094,585	1,491,117,637 (97.6)	–
Number of physicians with payments, n (%)	1251 (6.0)	1215 (5.8)	1310 (6.2)	1435 (6.8)	1416 (6.8)	1505 (7.2)	1426 (6.8)	1357 (6.5)	2757 (13.1)	2.1 (1.3 – 2.9)*
Payments per physician, $^a,b^										
Median (IQR)	28,053 (7,139–115,306)	26,724 (6,824–102,184)	30,910 (9,297–97,393)	37,190 (9,444–113,905)	38,002 (11,058–108,162)	38,271 (11,468–133,442)	34,536 (9,126–122,523)	29,067 (7,802–94,266)	85,080 (19,899–382,206)	-2.6 (-5.1 – 2.1)
Average (SD)	148,509 (565,878)	145,684 (628,210)	161,519 (837,801)	142,920 (539,345)	116,188 (298,841)	138,860 (363,418)	130,466 (371,556)	112,081 (493,591)	540,848 (2,309,907)	

Each year, 1.2% to 2.2% of gastroenterologists received direct research payments, while 5.8% to 7.2% received associated research payments. There were no consistent trends in the per-physician amounts of direct research and grant payments and associated research payments during the same period. The median annual associated research payments ranged from $26,724 to $38,271. However, the number of gastroenterologists receiving associated research payments significantly increased, from 1,251 in 2014 to 1,357 in 2021, reaching a peak of 1,505 in 2019. The relative annual average percentage change in the number of gastroenterologists receiving associated research payments was 2.1% (95% CI: 1.3%-2.9%, p<0.001) between 2014 and 2021 (Table 1).

Out of the associated research payments, only 2.2% ($33.2 million) was allocated for preclinical research. In contrast, 24.5% of the associated research payments were directed towards registered clinical trials (Table 2). The proportion of research payments for registered clinical trials increased from 14.0% in 2014 to 45.6% in 2021. The payment amounts for registered clinical trials rose by 165.7% from 2014 to 2021, increasing from $26.1 million to $69.3 million. Among the top 20 registered clinical trials with the highest associated research payments, there were 16 randomized controlled trials, 14 double-blind trials, and 12 trials focused on ulcerative colitis and/or Crohn's disease. The number of participants in these trials ranged from 177 to 10,078. Among the 284 companies making research payments to gastroenterologists, Gilead Sciences provided the largest amount, totaling $253.6 million, followed by AbbVie ($224.2 million) and Pfizer ($117.9 million). The top 10 and top 20 companies accounted for 68.0% and 83.2% of all research and grant payments, respectively.

**Table 2:** Associated research payments to preclinical trials and registered clinical trials between 2014 and 2021 Percentage of research payments is relative to the total amount of associated research payments for each year or overall.

Year	Payments to preclinical trials (%)^a^, $	Payments to registered clinical trials	
		Payment amounts (%)^a^, $	Number of clinical trials, n
2014	4,236,601 (2.3)	26,102,132 (14.0)	114
2015	5,608,733 (3.2)	24,139,114 (13.6)	117
2016	4,799,019 (2.3)	30,818,695 (14.6)	135
2017	2,029,356 (1.0)	28,874,960 (14.1)	160
2018	1,992,856 (1.2)	45,295,389 (27.5)	135
2019	5,385,369 (2.6)	71,808,850 (34.4)	143
2020	3,026,593 (1.6)	68,622,026 (36.9)	121
2021	6,097,548 (4.0)	69,345,436 (45.6)	155
Overall	33,176,075 (2.2)	365,006,603 (24.5)	505

## Discussion

This cross-sectional analysis of the Open Payments Database examined the whole size and fraction of research payments and grants to all gastroenterologists and hepatologists from the healthcare industry in the United States. This study found that more than $1.5 billion was either directly or indirectly allocated to gastroenterologists. Only 15.9% of gastroenterologists received these research payments over the eight years. Additionally, there was a significant increase in the number of gastroenterologists receiving research payments, with payments for registered clinical trials more than doubling over this period. These trials were generally rigorously designed and involved a large patient cohort, primarily aiming for new drug approvals or expanded drug indications. However, a relatively small fraction of these research payments was directed towards preclinical trials. 

In contrast to the study by Ying et al. [3], the research payments to gastroenterologists in this analysis were 55.5 times larger ($27.5 million vs. $1.5 billion). Payments provided to teaching hospitals and research institutions, where gastroenterologists served as principal investigators, were 41.4 times greater than those directly distributed to individual gastroenterologists. This difference in methodology would be one of the largest reasons for the difference in estimated amounts of research payments to gastroenterologists between this study and the previous study by Ying et al. These substantial investments by the healthcare industry in industry-sponsored clinical trials have undoubtedly been pivotal in the development of novel drugs and have significantly impacted patient treatment and quality of life within the field of gastroenterology [1,2]. 

This study found that only 15.9% of all gastroenterologists and hepatologists had the opportunity to receive research payments and grants from the healthcare industry over the eight-year period. When compared to previous studies using similar methodologies, a higher proportion of gastroenterologists and hepatologists received research payments than physicians in other specialties. For instance, the proportions of physicians receiving research payments were approximately 1.2% in emergency medicine [13], 7% in urology [24], 11% in neurosurgery [6], 12% in nephrology [10], 13% in endocrinology [11], 14% in pulmonology [4], and 15.0% in infectious diseases [8], while 20.2% in rheumatology [9]. Moreover, the per-physician annual amounts of associated research payments were moderate when compared to other specialties, ranging from $26,724 to $38,271. Median annual associated research payments were $27,409 to $36,700 for nephrologists [10] and $28,450 to $39,706 for pulmonologists [4], while $30,483 to $55,252 for infectious disease physicians [8], $39,138 to $61,620 for allergists [7], $40,300 to $66,163 for endocrinologists [11], and $40,527 to $64,023 for rheumatologists [9]. Thus, fortunately, it is unlikely that research payments and grants from the healthcare industry to specialists in gastroenterology and hepatology are significantly lower or higher than those for other medical specialties in the United States.

The observed figures may be attributed to the increased number of novel biologics and drugs for inflammatory bowel disease and hepatitis C virus infections. For instance, Gilead Sciences, the leading pharmaceutical company in research payments, has developed and manufactured several innovative drugs, including sofosbuvir (Sovaldi), ledipasvir-sofosbuvir (Harvoni), and velpatasvir-sofosbuvir (Epclusa). The healthcare industry predominantly directs its research payments toward conducting clinical trials to assess the safety and efficacy of medical products, particularly to obtain drug approvals or expand their indications to other diseases. Meanwhile, previous studies showed that there were fewer research payments to physicians from the healthcare industry in other specialties, such as emergency medicine, urology, and neurosurgery, where there were fewer novel drugs [6,13,24]. Thus, the large research funding for gastroenterologists and hepatologists could have been supported by the growing number of novel and effective drugs in the fields of gastroenterology and hepatology. 

Meanwhile, it should be noted that physicians participating in industry-sponsored trials and receiving research funding may receive substantial financial and non-financial personal incentives, such as promotions, increased professional recognition, and increased revenues. Furthermore, conflicts of interest are independently associated with more positive interpretations in industry-sponsored clinical trials [25]. In light of these considerations, it is imperative that all gastroenterologists maintain transparency regarding their financial relationships with the healthcare industry and understand the potential impact on their practice.

Limitations

The limitations of this study include the possibility of errors in public databases and unmeasured confounding factors that may influence payment trends. Additionally, research payments are primarily made to institutions and teaching hospitals for research that is conducted by physicians. However, the Open Payments Database does not provide information on how these payments were internally distributed among physicians and staff within these institutions and hospitals. Additionally, the Open Payments Database allows healthcare companies to delay in the submission of their research payments to the Centers for Medicare and Medicaid Services for up to four years. Therefore, there might be delayed, undisclosed research payments between 2018 and 2021 in this study.

## Conclusions

The healthcare industry has allocated substantial amounts of research funding to gastroenterologists and hepatologists. Furthermore, there was a significant increase in the number of these specialists receiving research payments over the eight-year period from 2014 to 2021 in the United States. Although only 15.9% of gastroenterologists and hepatologists received research payments, this proportion is consistent with figures reported in other internal medicine subspecialties. This study highlights the pivotal role that industry-sponsored clinical trials play in advancing research and treatments in the field of gastroenterology.
